# Geo-spatial high-risk clusters of Tuberculosis in the global general population: a systematic review

**DOI:** 10.1186/s12889-023-16493-y

**Published:** 2023-08-19

**Authors:** Titilade Kehinde Ayandeyi Teibo, Rubia Laine de Paula Andrade, Rander Junior Rosa, Reginaldo Bazon Vaz Tavares, Thais Zamboni Berra, Ricardo Alexandre Arcêncio

**Affiliations:** Department of Maternal-Infant and Public Health Nursing, Ribeirão Preto College of Nursing, University of São Paulo, Ribeirão Preto, Sao Paulo, Brazil

**Keywords:** Tuberculosis, High-risk, Spatial analysis, High-risk cluster, Spatiotemporal analysis

## Abstract

**Introduction:**

The objective of this systematic review is to identify tuberculosis (TB) high-risk among the general population globally. The review was conducted using the following steps: elaboration of the research question, search for relevant publications, selection of studies found, data extraction, analysis, and evidence synthesis.

**Methods:**

The studies included were those published in English, from original research, presented findings relevant to tuberculosis high-risk across the globe, published between 2017 and 2023, and were based on geospatial analysis of TB. Two reviewers independently selected the articles and were blinded to each other`s comments. The resultant disagreement was resolved by a third blinded reviewer. For bibliographic search, controlled and free vocabularies that address the question to be investigated were used. The searches were carried out on PubMed, LILACS, EMBASE, Scopus, and Web of Science. and Google Scholar.

**Results:**

A total of 79 published articles with a 40-year study period between 1982 and 2022 were evaluated. Based on the 79 studies, more than 40% of all countries that have carried out geospatial analysis of TB were from Asia, followed by South America with 23%, Africa had about 15%, and others with 2% and 1%. Various maps were used in the various studies and the most used is the thematic map (32%), rate map (26%), map of temporal tendency (20%), and others like the kernel density map (6%). The characteristics of the high-risk and the factors that affect the hotspot’s location are evident through studies related to poor socioeconomic conditions constituting (39%), followed by high population density (17%), climate-related clustering (15%), high-risk spread to neighbouring cities (13%), unstable and non-random cluster (11%).

**Conclusion:**

There exist specific high-risk for TB which are areas that are related to low socioeconomic conditions and spectacular weather conditions, these areas when well-known will be easy targets for intervention by policymakers. We recommend that more studies making use of spatial, temporal, and spatiotemporal analysis be carried out to point out territories and populations that are vulnerable to TB.

**Supplementary Information:**

The online version contains supplementary material available at 10.1186/s12889-023-16493-y.

## Introduction

Mycobacterium tuberculosis, which causes tuberculosis (TB), typically affects the lungs (pulmonary TB) but can sometimes affect other organs (extra pulmonary TB). It is primarily transmitted through coughing by bacilliferous individuals, contributing to the high incidence of the disease [1].

TB was a leading cause of death globally, until the appearance of the new coronavirus, responsible for the pandemic starting in 2020. TB is an infectious and communicable disease that primarily affects the lungs. About 6 million new cases are reported annually worldwide, leading to more than one million people dying annually. The emergence of acquired immunodeficiency syndrome (AIDS) and the emergence of drug-resistant TB further aggravate this scenario [2]. The disease remains an important global public health problem. Its relevance and magnitude can be evidenced by the estimates of the World Health Organization (WHO) with 8.7 million new cases and 1.4 million deaths per year [2].

Long latency period is very common among a large number of those infected with TB, with a lifetime risk of 10–15% eventually evolving to active disease. Only around 5% of those infected develop an active disease within the first two years. Individual immunological status has an impact on the likelihood of progression, and immunologically weakened patients have a considerably higher risk [3].

Years of progress in decreasing the burden of TB disease and providing crucial TB services have been reversed by the coronavirus disease 2019 (COVID-19) epidemic. Despite a few notable national and regional successes, the global TB targets are generally off course.

Reduced access to TB diagnosis and treatment has resulted in an increase in TB deaths. Best estimates for 2020 are 1.3 million TB deaths among HIV-negative people (up from 1.2 million in 2019) and an additional 214 000 among HIV-positive people (up from 209 000 in 2019), with the combined total back to the level of 2017. Declines in TB incidence (the number of people developing TB each year) achieved in previous years have slowed almost to a halt. These impacts are forecast to be much worse in 2021 and 2022 [4].

Despite being curable, TB is still ravaging throughout the entire world. Actions to mitigate and reverse these impacts are urgently required. The immediate priority is to restore access to and provision of essential TB services such that levels of TB case detection and treatment can recover to at least 2019 levels, especially in the most badly-affected countries.

In recent years, the discovery of infectious disease high-risk has been done using spatial–temporal cluster analysis [5]. Significant results were obtained when it was effectively applied to the identification of TB clusters [6]. According to prior research, TB is an infectious disease that spreads through the air and has a spatially and temporally heterogeneous distribution. It is thought that a better understanding of the spatial epidemiology of TB will help policymakers and other stakeholders develop effective regional prevention and control strategies [7].

Spatial analysis (SA) as a method involves the description of geographically cumulated TB data with respect to demographic, environmental, infectious and socioeconomic risk factors in a particular area. The SA of TB would give insight to the recent statistical update of the disease. Given the scenario that a low socio-economic status in a study area result in a high TB prevalence with a risk of TB, evaluations are needed to better understand the specific areas that are at high risk of TB and to make future predictions of likely occurrence or reoccurrence.

Studies that aim to examine the progression of the disease's symptoms over time benefit from the usage of databases. Based on continuous data streams, such studies are able to forecast the likelihood of incidents [8]. Additionally, the incorporation of various factors, particularly demographic and socioeconomic variables, in health research is made possible using geoprocessing techniques with geographic information systems (GIS) and spatial statistics [9], making it possible to generate hypotheses regarding the transmission of diseases in different populations [10].

The aim of this study therefore is to analyze studies that carried out geospatial analysis of TB through the instrumentality of databases to discover areas that are at high risk of TB (TB High-risk).

## Methods

Thus, this study seeks to advance knowledge in health surveillance of territories by filling a gap regarding spatial distribution of TB.

### Methods used for systematic mapping

The protocol of this review was registered on PROSPERO (CRD42021274287). This systematic review was developed in accordance with the Preferred Reporting Items for Systematic Review and Meta-Analysis Protocols (PRISMA-P) [11] recommendations which involved the following steps:

### Step 1: Research question

The research question was based on the “CoCoPop” strategy;“Where are the geo-spatial high-risk of tuberculosis among the general population located in any part of the globe?”, the acronym CoCoPop was structured as follows: Condition (Co) corresponding to TB; Context (Co) any part of the globe and Population (Pop) general population. The strategy is represented in Table 1.

**Table 1:** CoCoPop structure for research question

Description	Abbreviation	Research Components
Condition	Co	Tuberculosis
Context	Co	Global Context
Population	Pop	General population

Source: Prepared by the author

Thus, the main research question that guided this review was the following: Where are the specific territories and areas that are high in TB, identifiable as specific TB high-risk?

Secondary questions (Q) were established for a better characterization of the publications to be obtained; they include:

- Q1—Which countries have publications on geospatial analysis of TB?
- Q2—Which studies involve association with social vulnerability?
- Q3—What is the relationship between TB cases and their specific hotspot regions?
- Q4- Are there other prominent factors that affect hotspot’s location?

### Step 2: Research protocol

A research protocol was elaborated, in which all stages of the review process were established. Ecological, cross-sectional and observational studies that had in their scope the spatiotemporal analysis and geospatial analysis of TB were included for study in the protocol.

### Step 3: Search for evidence

#### Data base

The bibliographic databases MEDLINE (Medical Literature Analysis and Retrieval System Online) via PubMed (Public/Publisher MEDLINE) – United States National Library of Medicine – access via: https://www.ncbi.nlm.nih.gov/pubmed/; LILACS (Latin American Literature in Health Sciences (*Literatura Latino-Americana e do Caribe em Ciências da Saúde*)) via BVS (Virtual Health Library (*Biblioteca Virtual em Saúde*)) – access via: http://bvsalud.org/; EMBASE (Excerpta Medica Database) via CAFe (Federated Academic Community – *Comunidade Acadêmica Federada*) of CAPES (Coordination for the Improvement of Higher Education Personnel – *Coordenação de Aperfeiçoamento de Pessoal de Nível Superior*) journals portal – access via: https://www-periodicos-capes-gov-br.ezl.periodicos.capes.gov.br/index.php?; Scopus (SciVerse Scopus) via CAFe from CAPES journals portal; Web of Science (Web of Science Core Collection) via CAFe from CAPES journals portal; and Google Scholar – access via: https://scholar.google.com.br/?hl=pt. This is described in details in Table S1.

#### Search strategy

The search was carried out in February 2023 on PubMed, LILACS, EMBASE, Scopus (SciVerse Scopus) and Web of Science. For gray literature search, it was carried out with Google Scholar. The keywords and descriptors used for the search were identified in the Descriptors in Health Science (DeCS) and Medical Subject Headings (MeSH). The search strategies were adapted for each database using Boolean operators (OR and AND) as shown in Table S1. In the search, language limits will not be used.

#### Inclusion criteria

The languages included the study is English, during the initial search however, there was no language restriction once the article has versions in English even though published originally in another language. Studies which had their central object of study being geospatial and or spatial analysis of TB were included, scientific articles available in full and published between 2017 and 2023 were included, as well as studies conducted among the general population, there was no language restriction in the search.

#### Exclusion criteria

Studies that addressed specific groups, such as health workers, students, pregnant women, among others were excluded, we excluded studies that did not have human beings as the object of study, we also excluded Course Completion Works (TCC), simple and expanded abstracts, letters to the editor. Articles that were not available in English were excluded. Studies that include only latent infection with *Mycobacterium Tuberculosis* or only special populations like TB-coinfection or people deprived of liberty were not excluded.

#### Initial selection

The Qatar Computing Research Institute Rayyan Systematic Review application QCRI was used to manage citations chosen from databases [12]. From there, we excluded duplicate publications and titles and abstracts read by two independent reviewers. In case of any doubt or disagreement between the two reviewers regarding the inclusion of any material, a third reviewer was consulted. To confirm the inclusion of selected articles, all eligible articles were read in full.

The entire search process and the eligibility process of the materials initially found and finally included were presented in a flow diagram, as recommended by the PRISMA [13].

#### Final extraction and selection

Data was extracted using a standard form designed by the research team, after which an independent pair of trained reviewers compared the results and, in the event of dispute, a third reviewer was consulted. The data extraction form used was prepared according to items created in consonance by the authors, they are: Authors, Year of publication, Country of study, Purpose of study, Type of data, Type of cases, Geographical level, Spatial methods, Cluster detection methods, Statistical regression methods, Spatial smoothing techniques, Study period, Results, Tuberculosis high-risk, Hotspot characteristics.

### Summary of studies

The studies included in the final selection were cataloged, so that the mapping results could be better visualized and understood. A table with the summary of the final articles was built containing an identification code to facilitate reference to the studies listed throughout the text, the Study, Authors / Year of publication / Country of study, Study Objective, Study period, Data type / Geographic level, Case type, Study, Type of map, Cluster detection method, Regression statistics method, smoothing technique and results were used. This is represented in Table S2 and S3.

### Summary of systematic mapping results

**Fig. 1:**
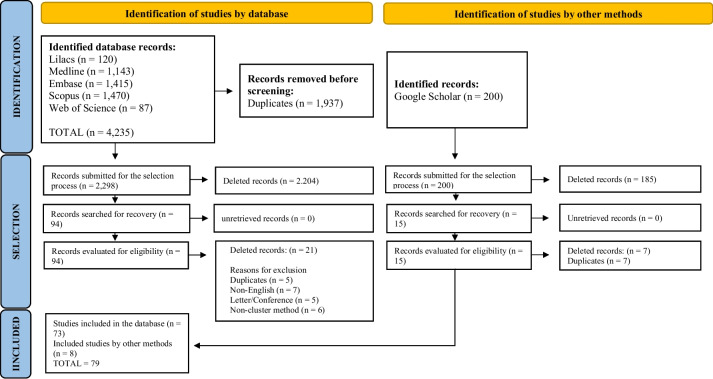
PRISMA diagram showing article selection. Source: Modified from [13] Page et al. (2021)

Systematic mapping was performed according to the previously described protocol. In the search carried out in the databases, 4,235 publications were found, while on Google Scholar, we found 200 articles. out of this 200, 102 publications remained in the study but the 23 studies that did not meet the inclusion criteria after reading all the full text were excluded leaving 79 studies. Figure 1. illustrates the steps followed to conduct the mapping with the respective result.

## Results

Table S2 presents the authors, locations where the studies were carried out, which cut across Africa, North and South America and Asia. About 90% of the studies used notification data and the general study period ranged from 1982 till date. More than 50% of the study used pulmonary and extrapulmonary cases in their study.

Table S3 elucidates methods used to identify the high-risk, majority used spatial scan statistic, generated thematic maps and used a specific regression model. 80% of the high-risk identified were associated with poor socioeconomic status and overcrowding.

The Table S4 displays the characteristics of the high-risk obtained from the study. Socioeconomic condition, overpopulation, climate and border-sharing all determined high-risk clustering.

**Fig. 2:**
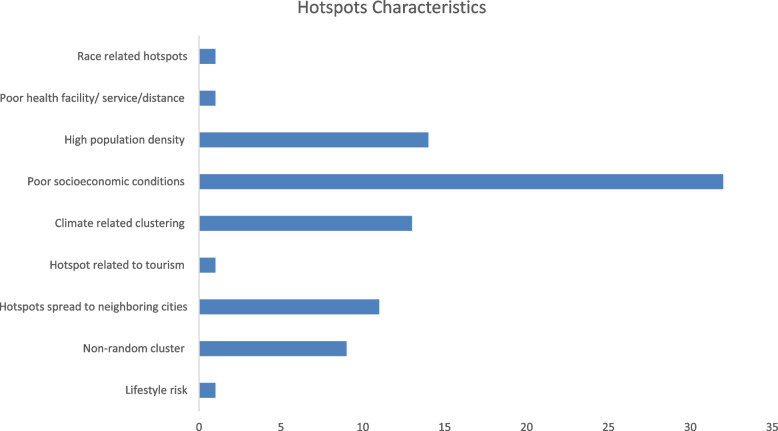
Characteristics of High-risk. Source: Prepared by author

Figure 2 represents the TB distribution rate included in the study based on specific factors. The information is obtained from Table S4 as showed.

**Fig. 3:**
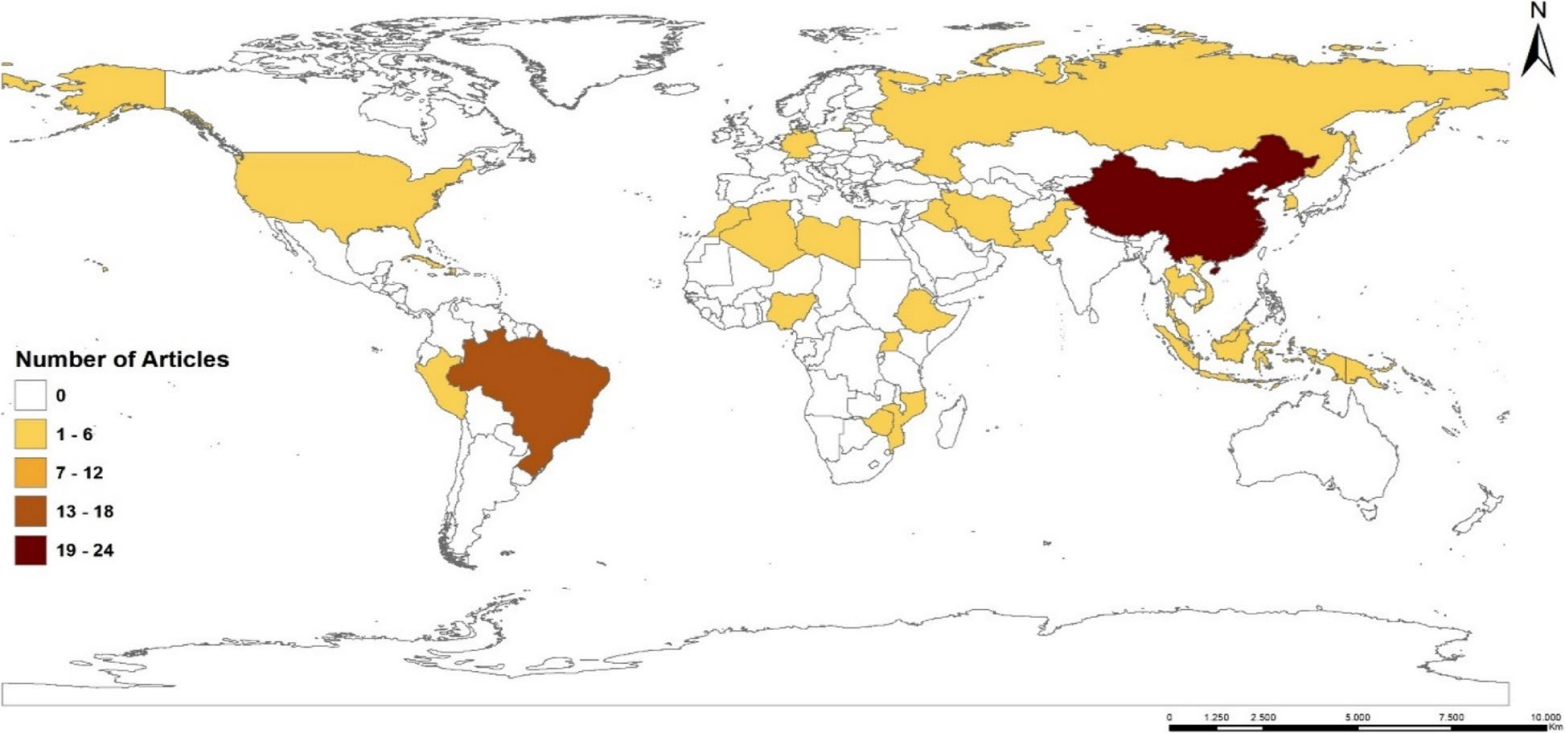
Distribution of countries included in the study. Source: Prepared by author

Figure 3 represents the distribution of countries that performed geospatial analysis of TB included in the study.

## Discussion

The aim of this systematic review was to identify TB high-risk among the general population globally and this can contribute to reducing TB incidence and mortality and thus help channel resources and efforts to this region [4]. A total of 79 published articles which study period spans between 1982 and 2022 which is a 40 years study period were evaluated.

The characteristics of the high-risk and the factors that affects hotspot’s location is evident through studies related to poor socioeconomic conditions constituting (39%), followed by high population density (17%), climate related clustering (15%), high-risk spread to neighboring cities (13%), unstable and non-random cluster (11%) (Fig. 2).

To answer these questions above, out of the 79 studies, China has the highest number of studies constituting 40% of all countries that have carried out geospatial analysis of TB followed by Brazil with 23%, Iran and Ethiopia both have 6% and others 2% and 1% (Fig. 3).

From studies in China, TB high-risk was suggested to have been imported from nearby countries with a high incidence of the disease in one of the studies, in the same study, it was inferred that the high-high areas spread from the northeastern to the southeastern region, showing the way TB burdens possibly spread between territories especially those lying close to each other. The United Nations gave a supporting report about this, evidencing it is difficult to track TB cases as it moves across borders [14] also found that TB as an epidemic was spread between the borderland of Myanmar and Thailand due to mobility of migrants and microbes.

Also, in another China-based study, it was found that the hotspot location discovered was as a result of underdeveloped economic conditions, inefficient healthcare, and a lack of awareness of essential prevention knowledge as evidenced by TB report of the World health organization, Tuberculosis is a disease closely associated with poverty and poor living conditions [4].

From studies in Brazil, high-risk regions discovered were among the population composed of individuals with difficulties in accessing health services and are therefore more deprived. This population is precisely the one that lives in places of difficult localization especially in the Northeastern region [15] this is on par with the China based study as well. The localization of TB in the Northwestern part of the country proves both socioeconomic relationship of TB with poverty as well as the specificity of TB to particular locations as evidenced by a study by [15] who found out that in the period from 2010 to 2019 (prior to the COVID-19 pandemic), the country and all of its macro-regions showed an increasing temporal trend of the TB notification rate, with an emphasis on the Northern Region of Brazil.

From studies in Ethiopia, Generally, the TB high-risk cluster in the country over the study period appeared in rural Ethiopia and in more urbanized areas such as Addis Ababa, and other parts of Northwest Ethiopia further emphasizing the localization of TB high-risk, effect of high population density and poor socioeconomic condition on TB. The study also recommended that ensuring access to TB diagnosis and treatment could aid in TB incidence diminution, improve adherence to therapy, and strengthen the community's TB control program [16].

The result points to the presence of vulnerable populations in specific territories affecting people of specific socioeconomic status (social determinants of health). This points to specific locations having high TB prevalence and this is in support [17] that evidenced that TB burden was associated with specific communities, evidencing three high-risk detected in the Northwest, Northcentral and Metropolitan regions. In this sense, the high-risk were concentrated in the northern region (66.7%) similar to our study.

TB is a disease prevalent among the poor and caused majorly by poor living conditions, higher in places of high population density with difficulty in accessing health services. The population being poor goes from socioeconomically bad to worse following the occurrence of TB, as the total cost of TB treatment is greater than 20% annual household income in these populations [4]. The result of this study confirms this evidence showing that high TB incidence which reflect as clusters (high-risk) are due to of low socioeconomic conditions and poor health care access.

Comprehensive care can be best given to patients through greater resource utilization, active diagnosis, and affordable medications. In addition, all of these require research to offer an informed platform for prudent use of the resources that are already available. Discovery of high-risk and vulnerable populations and communities will help to put the limited resources available to optimum use.

The End TB Strategy of the WHO outlines global goals and targets for significant decreases in the annual number of TB deaths between 2016 and 2035. The first goal is a decrease of 35% from 2015 to 2020. Following plans for reductions of 90% by 2030 and 95% by 2035, the next milestone in 2025 is a 75% decrease from 2015 levels [4]. It is necessary to accomplish worldwide milestones and targets for reducing the number of people who contract TB each year in order to meet these targets. The slight increase in the mortality rate observed could be a sequel to this set target by the WHO in a bid to reduce the burden. Improved case reporting and surveillance would increase the number of cases reported and point out the fatality of TB for a more effective control.

It is known that TB is a disease closely related to the social determinants of health and is associated with low social class, poverty, social vulnerability and immunosuppressive diseases such as HIV can influence the appearance of the disease in which the person, in addition to having who deal with the disease itself, usually also suffer from stigma from the society in which they are inserted, usually influenced by the perception of risk or lack of knowledge about the disease on the part of the community.

TB with its symptoms and the consequent stigma doubly influence the impact on TB control, since the respiratory symptomatic person, who has a long-lasting cough, has reservations about seeking a health service for fear of the diagnosis. In addition, after the TB diagnosis has been made, given that the dread of being discovered as having the TB bacillus would make other elements of a person´s life problematic, it is more difficult to continue with the care [18]. This behavior is observed even in countries that have a low incidence of TB, since stigma is one of the most striking characteristics when we talk about TB.

This fear of seeking medical care and consequent delay in diagnosis makes the person spend more time in the bacilliferous phase, that is, contaminating other people, and thus makes it difficult to break the TB transmission chain, in addition to being able to take the person infected to serious complications that may require hospitalization or even death due to the delay in being diagnosed and starting treatment.

TB has in addition to the disease itself, the stigma that people with TB suffer can cause very harmful consequences in their daily lives, such as low self-esteem, insults, ridicule, self-exclusion and social exclusion, leading to a worsening of their quality of life and social status, as identified in a study conducted in Zambia.

The use of georeferencing techniques provides an overview of the areas that should be prioritized in the fight against TB and deserve attention from the perspective of the social determinants of health in the health-disease process. Alleviating the problems of stigmatization. The identification of areas considered at risk or with a higher concentration of TB cases is extremely important for the planning and implementation of public policies and strategic actions to prevent transmission and timely diagnosis of the disease, with emphasis on actions to actively search for symptomatic patients breathing.

With the identification of critical areas for the analyzed event, where the risk of TB transmission is more intense, priority should be given to investigating and monitoring risk factors for TB infection in these areas, with the aim of helping TB programs. prevention in the effective control of both diseases, since the dynamics of disease transmission is not limited to political-administrative borders. It is important that managers prioritize the organization of the health care network throughout the territory in order to facilitate access and early diagnosis of TB. In addition, the importance of rapid initiation of treatment and the correct drug regimen according to the form of TB diagnosed is highlighted, which should be started as soon as possible.

Therefore, it is important for managers at all levels of administration to focus on combat strategies aimed at early diagnosis and active search for cases. In addition, with the aim of improving the epidemiological indicators of both diseases, a necessary and effective tool is the periodic training of health professionals, not only in the biological sense, but also to understand the epidemiological and social reality of the territory in which they are inserted, so that they can understand the relationship between TB and the social determinants of health. This study therefore contributes to knowledge by pointing out vulnerable populations for preferential application of TB control policy.

The review largely confirmed many general characteristics previously observed on the inter relationship between Tuberculosis and poor socio-economic conditions, precarious living conditions and overcrowding., but also documented the extensive methodological limitations of studies that used varying methodologies that are divergent and heterogenous especially in terms of the population size and context which this present study evaluated 79 published articles [19–93] presented in Table S2 and S3. Most of the methods used in the studies reviewed are best suited for low/high endemic regions depending on the model applied., among other limitations. Notwithstanding, several consistent characteristics of TB in different population scenario were identified which could give insight to critical areas for the analyzed event i.e., where the risk of TB transmission is more intense.

## Conclusion

The study shows the spatial analysis of event, in the locations examined, given that it follows the opposite direction to the policies aimed at eliminating TB through the End TB Strategy, thus, the attention of the health authorities becomes necessary, given that the policies and actions are not being effective, mainly in the hot areas identified in the study- characterized majorly by poor socioeconomic conditions and high population density. We have been able to confirm that there exists specific high-risk for TB which are areas that are related to low socioeconomic conditions, over-population and spectacular weather conditions, these areas when well-known will be easy targets for intervention by policy makers We recommend that more studies making use of spatial, temporal and spatio-temporal analysis be carried to point out territories and populations that are vulnerable to TB. Although our study aimed to analyze TB burden on a global basis, only few studies are available and have covered few parts of the globe.

### Supplementary Information

**Additional file 1.**

## Data Availability

The datasets used and/or analyzed during the current study available from the corresponding author on reasonable request.
