# Supplementary material for: Geo-spatial high-risk clusters of Tuberculosis in the global general population: a systematic review
Source: BMC Public Health. 2023 Aug 19;23:1586. doi: 10.1186/s12889-023-16493-y (PMC10439548; doi:10.1186/s12889-023-16493-y)
Supplement: Supplementary file 1 — Additional file 1. [file 12889_2023_16493_MOESM1_ESM.docx]

**Supplementary Tables**

**Table S1 -** Article search strategies used to carry out systematic review on geo-spatial hotspots of tuberculosis in the general population globally.

| **Databases** | **Vocabulary found/free vocabulary** |
| --- | --- |
| **MEDLINE** | ("tuberculosi" OR "tuberculosis"[MeSH Terms] OR "tuberculosis" OR "tuberculoses" OR "tuberculosis s" OR "tb" AND ("hotspot" OR "hotspots" OR "hot-spot" OR "hot-spot" OR ("hotspot" OR "hotspots" OR "hot-spots" OR "hot-spots" OR (("geographic" OR "geographical" OR "geographically" OR "geographics" AND ("focal" OR "focalities"[All Fields] OR "focality"[All Fields] OR "focalization"[All Fields] OR "focalized" OR "focally" OR "focals" OR "local" OR "localisation" OR "localisations" OR "localise" OR "localised" OR "localises" OR "localising" OR "localization" OR "localizations" OR "localize" OR "localized" OR "localizer" OR "localizers" OR "localizes" OR "localizing" OR "locally" OR "locals" AND ("risk"[MeSH Terms] OR "risk" OR "risk area"] OR "risk areas" OR "spatial analysisOR "spatial interpolation" OR "spatial dependency" OR "spatial dispersion" OR "spatiotemporal analysis" OR "spatio-temporal analysis" OR "spatiotemporal patterns" OR "spatio-temporal patterns" OR "spatiotemporal epidemiology" OR "spatio-temporal epidemiology" OR "geographic information systems" OR "medical topography" OR ("geographic mapping"[MeSH Terms] OR ("geographic" AND "mapping" OR "geographic mapping" OR "georeferencing" OR "georeferenced" OR "geoprocessing" |
| **LILACS** | (tuberculose OR tuberculosis OR tb) AND (hotspot OR "hot-spot" OR "hot spot" OR hotspots OR "hot-spots" OR "hot spots" OR "geographical localization of risk" OR "risk area" OR "risk areas" OR "spatial analysis" OR "spatial interpolation" OR "spatial dependency" OR "spatial dispersion" OR "spatiotemporal analysis" OR "spatio-temporal analysis" OR "spatiotemporal patterns" OR "spatio-temporal patterns" OR "spatiotemporal epidemiology" OR "spatio-temporal epidemiology" OR "geographic information systems" OR "medical topography" OR georeferencing OR geoprocessing OR "punto alto" OR "localização geográfica de risco" OR "área de risco" OR "áreas de risco" OR "localización geográfica de riesgo" OR "zona de riesgo" OR "zonas de riesgo" OR "análise espacial" OR "interpolação espacial" OR "dependência espacial" OR "dispersão espacial" OR "análise espaço-temporal" OR "padrões espaço-temporal" OR "epidemiologia espaço-temporal" OR "análisis especial" OR "interpolación espacial" OR "dependencia espacial" OR "dispersión especial" OR "análisis espaciotemporal" OR "patrones espacio-temporales" OR "epidemiología espaciotemporal" OR "sistemas de informação geográfica" OR "sistemas de información geográfica" OR "topografia médica" OR "topografía médica" OR georreferenciamento OR georeferenciación OR geoprocessamento OR geoprocesamiento) |
| **EMBASE** | #1 'tuberculosis'/exp OR tuberculosis OR 'tb'/exp OR tb  #2 'hotspot'/exp OR hotspot OR 'hot-spot' OR 'hot spot'/exp OR 'hot spot' OR hotspots OR 'hot-spots' OR 'hot spots' OR 'geographical localization of risk' OR 'risk area' OR 'risk areas' OR 'spatial analysis'/exp OR 'spatial analysis' OR 'spatial interpolation' OR 'spatial dependency' OR 'spatial dispersion' OR 'spatiotemporal analysis'/exp OR 'spatiotemporal analysis' OR 'spatio-temporal analysis'/exp OR 'spatio-temporal analysis' OR 'spatiotemporal patterns' OR 'spatio-temporal patterns' OR 'spatiotemporal epidemiology' OR 'spatio-temporal epidemiology' OR 'geographic information systems'/exp OR 'geographic information systems' OR 'medical topography'/exp OR 'medical topography' OR 'georeferencing'/exp OR georeferencing OR geoprocessing  #3 #1 AND #2 |
| **Scopus** | TITLE-ABS-KEY (tuberculosis OR tb ) AND TITLE-ABS-KEY ( hotspot OR "hot-spot" OR "hot spot" OR hotspots OR "hot-spots" OR "hot spots" OR "geographical localization of risk" OR "risk area" OR "risk areas" OR "spatial analysis" OR "spatial interpolation" OR "spatial dependency" OR "spatial dispersion" OR "spatiotemporal analysis" OR "spatio-temporal analysis" OR "spatiotemporal patterns" OR "spatio-temporal patterns" OR "spatiotemporal epidemiology" OR "spatio-temporal epidemiology" OR "geographic information systems" OR "medical topography" OR georeferencing OR geoprocessing) |
| **Web of Science** | (TS=(tuberculosis OR tb)) AND TS=(hotspot OR "hot-spot" OR "hot spot" OR hotspots OR "hot-spots" OR "hot spots" OR "geographical localization of risk" OR "risk area" OR "risk areas" OR "spatial analysis" OR "spatial interpolation" OR "spatial dependency" OR "spatial dispersion" OR "spatiotemporal analysis" OR "spatio-temporal analysis" OR "spatiotemporal patterns" OR "spatio-temporal patterns" OR "spatiotemporal epidemiology" OR "spatio-temporal epidemiology" OR "geographic information systems" OR "medical topography" OR georeferencing OR geoprocessing) |
| **Google Scholar (Two strategies was used)** | spatiotemporal analysis tuberculosis  hotspots tuberculosis |

**Table S2 -** Characteristics of the studies included in the systematic review of the geo-spatial hotspots of tuberculosis in the global general population.

| **Study** | **Authors / Year of publication / Country of study** | **Study Objective** | **Study period** | **Data type / Geographic level** | **Case type** |
| --- | --- | --- | --- | --- | --- |
| **S1**  **(19)** | RIDZUAN et al., 2021  Malaysia | Utilizing a public participatory gis (ppgis) technique and a 5-risk scale from 1 to 5 to study the effects of lifestyle risk variables on TB cases in Shah Alam. | 2017 | Survey / Region | Lifestyle risk factors of TB diseases |
| **S2**  **(20)** | SELMANE; L'HADJ, 2021  Algeria | Create a forecasting model, define the spatiotemporal distribution, and determine the seasonal pattern of tuberculosis (TB) in Algeria. | 1982 to 2019 | Notification / Country | Pulmonary and extra pulmonary |
| **S3**  **(21)** | MESQUITA et al., 2021  Brazil | To present the geographic and epidemiological trends of TB infections between 2013 and 2018 in the Pará, Brazil, Amazonian area, Marajó Island. | 2013-2018 | Notification / Region | Pulmonary and extra pulmonary |
| **S4**  **(22)** | TANJUNG et al., 2021  Indonesia | To identify the prevalence of the pulmonary Tb risk factors in the Kabanjahe District, Karo Regency. | Jan-Oct 2020 | Survey / Region | Pulmonary and extra pulmonary |
| **S5**  **(23)** | YU et al., 2020  China | To evaluate the temporal and spatial distribution characteristics of PTB in Chongqing. | 2011-2018 | Notification / City | Only Pulmonary TB |
| **S6**  **(24)** | XIA et al., 2020  China | to describe the case notification rate (CNR) and determine what factors influence the TB pandemic. | 2006 to 2015 | Notification / Province | Only Pulmonary TB |
| **S7**  **(25)** | CHIRENDA et al., 2020  Zimbabwe | To use geospatial methods to describe the presence of hotspot transmission of TB cases in Harare city between 2011 and 2012. | 2011 and 2012 | Notification / City | Not specified (TB cases) |
| **S8**  **(26)** | PEREIRA et al., 2022  Brazil | To study the temporal trend of Santa Catarina's tuberculosis incidence rate by sex from 2010 to 2019 and to define the profile of patients. | 2010-2019 | Notification / State Level | Not specified (New TB cases) |
| **S9**  **(27)** | CHEN et al., 2019  China | To determine the spatial-temporal dynamic process and high-risk area of reported cases of pulmonary tuberculosis (PTB), sputum smear-positive tuberculosis (SSP-TB), and sputum smear-negative tuberculosis (SSN-TB) in Yunnan, western China, from 2005 to 2018. | 2005 to 2018 | Notification / Province | Pulmonary and extra pulmonary |
| **S10**  **(28)** | YANG et al., 2019  China | To look into the spatiotemporal distribution of TB at the township level throughout urbanization in the brand-new urban area of Nanchang. | 2010 to 2018 | Notification / Region | Not specified (TB data) |
| **S11**  **(29)** | LI et al., 2019  China | From 2009 to 2015, investigate the spatiotemporal distribution of TB and its contributing factors in China's mainland. | 2009-2015 | Notification / Region | Pulmonary and extra pulmonary |
| **S12**  **(30)** | GEHLEN et al., 2019  Brazil | In a Brazilian city with a high TB incidence, to elucidate the prevalence of TB and its associations with the Human Development Index (HDI). | 2011-2013 | Notification / City | Pulmonary and extra pulmonary |
| **S13**  **(31)** | ZHANG et al., 2019  China | To identify the geographic distribution of tuberculosis and its relationship to environmental factors in mainland China. | 2005-2015 | Notification / Country | Pulmonary and extra pulmonary |
| **S14**  **(32)** | ZHANG et al., 2018  China | To investigate the factors influencing the prevalence of tuberculosis and the temporal and geographic relevance of the pulmonary tuberculosis pathogenesis. | 2008-2015 | Notification / Country | Only Pulmonary |
| **S15**  **(33)** | LIU et al., 2018  China | To examine the spatial and temporal clustering analysis of tuberculosis at the prefecture level in mainland China from 2005 to 2015. | 2005-2015 | Notification / Country | Pulmonary and extra pulmonary |
| **S16**  **(34)** | RODRIGUES et al., 2017  Brazil | To examine the geographic distribution of the endemic tuberculosis from 2002 to 2011 in Rio de Janeiro State. | 2002 to 2011 | Notification / State | pulmonary and extra pulmonary |
| **S17**  **(35)** | KHAZAEI et al., 2019  Iran | To look at the regional and spatial distribution of TB in Hamadan Province from 1992 to 2013. | 1992-2013 | Notification / Country | Pulmonary and extra pulmonary |
| **S18**  **(36)** | FUSCO et al., 2017  Brazil | Analyzing the spatial distribution of patients in a municipality in the state of So Paulo and describing the epidemiological clinical picture of tuberculosis. | 2008-2013 | Notification / City | Pulmonary and extra pulmonary |
| **S19**  **(37)** | MAGALHÃES et al., 2017  Brazil | To use spatial statistical models to discover pertinent socioeconomic factors for the prevalence of the disease in order to analyze the spatial pattern of tuberculosis (TB) from 2005 to 2008. | 2005-2008 | Notification / Municipal | Pulmonary and extra pulmonary |
| **S20**  **(38)** | MOHAMMED et al., 2019  Iraq | Using geographic information science (GIS) technology to identify hotspot geographic locations with PTB incidence and to assess spatial global autocorrelation. | 2010-2016 | Notification / City | Only Pulmonary |
| **S21**  **(39)** | CHINPONG et al., 2022  Thailand | To outline the TB incidence and mortality patterns in Thailand from 2011 to 2020, including their temporal trends and geographic distribution. | 2011-2020 | Notification / Country | Pulmonary and extra pulmonary |
| **S22**  **(40)** | PUSCH et al., 2019  Germany | The locations with the highest densities of incident TB cases and variations in distribution over a ten-year period are identified in order to analyze the pattern of case distribution within the municipal limits. | 2006-2015 | Notification / Country | Pulmonary and extra pulmonary |
| **S23**  **(41)** | ANDRADE et al., 2021  Brazil | To locate the geographic areas of a Brazilian municipality that are more likely to experience tuberculosis and to have successful treatment. | 2013-2018 | Notification / City | Pulmonary and Extra pulmonary |
| **S24**  **(42)** | WANG et al., 2021  China | The Beijing-Tianjin-Hebei metropolitan agglomeration, which is a region with a high number of aging households and socio-economic inequality, was the focus of the first objective, which was to look into spatio-temporal variation in the incidence of TB among people older than 65 years. The second goal was to look at any potential non-linear relationships between socioeconomic characteristics and the risk of TB in this aging population. | 2009-2014 | Notification / Region | Only Pulmonary |
| **S25**  **(43)** | RENGGANIS WARDANI et al., 2020  Indonesia | To examine the temporal and spatial patterns of TB clusters in Bandar Lampung, Indonesia, from 2015 to 2016, and to pinpoint the clusters' features for population density and percentage of poverty. | 2015 to 2016, | Survey City | Pulmonary and extra pulmonary |
| **S26**  **(44)** | SATRIANI et al., 2018  Indonesia | To identify risk factors for TB illness occurrence in Barru regency and use geographic information systems (GIS) to map the geographical patterns of TB. | May-Jun 2016 | Survey / City | Only Pulmonary |
| **S27**  **(45)** | ZUO et al., 2020  China | To track the epidemiology and spatial-temporal aspects of tuberculosis in China from 2004 to 2017. | 2004- 2017 | Notification / Country | Pulmonary and extra pulmonary |
| **S28**  **(46)** | ASEMAHAGN et al., 2021  Ethiopia | Determine whether reported cases of pulmonary tuberculosis (PTB) in the East Gojjam Zone of northwest Ethiopia are geographically and chronologically clustered. | 2013-2019 | Notification / Region | Only Pulmonary |
| **S29**  **(47)** | SHOJAEI et al., 2017  Iran | To provide two cutting-edge statistical techniques, take into account the spatial distribution of tuberculosis incidence in Iran. | Not mentioned | Notification / Country | Only Pulmonary |
| **S30**  **(48)** | JIANG et al., 2022  China | To provide information on the spatiotemporal distribution traits of reported BP-PTB and notified BN-PTB in Northwest China in order to support interventions and enhance PTB management. | 2011-2018 | Notification / Province | Only Pulmonary |
| **S31**  **(49)** | DUAN et al., 2022  China | To identify the epidemic's features and the spatiotemporal distribution of TB cases reported in Shandong Province, and to offer a scientific foundation for the creation of more potent TB prevention and control measures. | 2016-2020 | Notification / City | Pulmonary and intrapulmonary |
| **S32**  **(50)** | ROMANYUKHA et al., 2020  Russia | Moscow's high-incidence residential regions for tuberculosis will be identified utilizing spatiotemporal analysis of incidence data. | 2000-2015 | Notification / City | Only Pulmonary |
| **S33**  **(51)** | SADEQ et al., 2018  Morocco | Analyzing TB trends in Morocco, locating TB geographical clusters, and identifying TB-related variables. | 1995-2014 | Notification / Country | Only Pulmonary |
| **S34**  **(52)** | CARRASCO-ESCOBAR et al., 2020  Peru | The spatial distribution and clustering of TB cases in Lima, Peru, as well as the co-occurrence of PM2.5 and economic index clusters, were determined. | 2015-2017 | Notification / Country | Only Pulmonary |
| **S35**  **(53)** | BROOKS et al., 2022  Peru | To detect local hot areas of rates of reported tuberculosis cases using regularly gathered data, geographic information, and census data. | 2013-2017 | Notification / Region, District | Only Pulmonary |
| **S36**  **(54)** | SHAWENO et al., 2018  Ethiopia | To comprehend the geographic spread of tuberculosis from hotspots to areas in a remote area of Ethiopia that are placed at various distances from one another. | 2010-2014 | Notification / City | Pulmonary and Extra pulmonary |
| **S37**  **(55)** | GWITIRA et al., 2021  Zimbabwe | Using GIS and spatial statistics to investigate the geographical patterns of TB recurrence at the district level in Zimbabwe from 2015 to 2018 in order to identify areas at higher risk for the prioritization of control and intervention measures | 2015-2018 | Notification / Country | Only Pulmonary |
| **S38**  **(56)** | GEMECHU; DEBUSHO, 2022  Ethiopia | To evaluate the spatial clustering of concurrent TB and HIV patients in Ethiopia at the district level. | 2015-2018 | Notification / Country | Pulmonary and extra pulmonary |
| **S39**  **(57)** | MOLLALO et al., 2019  USA | To investigate the geographic spread of the illness and the viability of MLTs in TB modeling under the following suppositions (1) All reported county-level TB incidence statistics reflect the prevalence of the disease across the US, and (2) environmental and socioeconomic factors have an impact on the risk of TB infection. | 2006-2010 | Notification / Country | Pulmonary and extra pulmonary |
| **S40**  **(58)** | IM et al., 2021  South Korea | To research TB cases in Korea from a socioeconomic and environmental vantage point. | 2008-2016 | Notification / Country | Pulmonary and Extra pulmonary |
| **S41**  **(59)** | GELAW et al., 2019  Ethiopia | The Amhara Region's sociodemographic characteristics of spatial clusters were described in order to determine the spatial distribution of TB and its prevalence. | 2014-2017 | Notification / Region | Pulmonary and Extra pulmonary |
| **S42**  **(60)** | MOHIDEM et al., 2021  Malaysia | To elucidate the geographic location of TB cases in the Gombak district and their relationship to sociodemographic and environmental parameters | 2013-2017 | Notification / Region / District | Pulmonary and Extra pulmonary |
| **S43**  **(61)** | ROBSKY et al., 2020  Uganda | To ascertain, based on routinely collected data, whether small-scale geographic areas which have elevated reports of TB rates have a proportionately high risk of undiagnosed prevalent TB. | 2018-2019 | Notification / City (community) | Only Pulmonary |
| **S44**  **(62)** | LI et al., 2022  China | To provide evidence for the creation of more sensible public health policies, as well as for the prevention and reduction of TB incidence. | 2010-2017 | Notification / Country | Only Pulmonary |
| **S45**  **(63)** | KHALIQ et al., 2022  Pakistan | To identify spatial-temporal TB clusters in Pakistan and comprehend patterns in TB notification throughout the country's various. | 2007-2020 | Notification / Region | Only Pulmonary |
| **S46**  **(64)** | HUANG et al., 2022  Peru | We find hotspots for local TB transmission by measuring the pairwise genomic distances between TB patient isolates as a function of geographic distance, and we contrast these locations with high-incidence hotspots in the same regions. | 2009-2012 | Survey / City | Only Pulmonary |
| **S47**  **(65)** | BERRA et al., 2022  Brazil | To display and categorize the time series of COVID-19, TB notification, cure, treatment abandonment, and mortality; to assess the influence of the new coronavirus pandemic on these indices in Brazil; and to assess the existence of geographical autocorrelation between TB and COVID-19. | 2010-2021 | Notification / City | Pulmonary and Extra pulmonary |
| **S48**  **(66)** | SOUSA et al., 2022  Brazil | To examine the spatial distribution of TB incidence and the factors that influence it. | 2001-2017 | Notification / region | Pulmonary and Extra pulmonary |
| **S49**  **(67)** | PAIVA et al., 2022  Brazil | To investigate the temporal, spatial, spatial-temporal, and chronological-spatial effects of socially vulnerable individuals on the incidence of tb in Brazil from 2001 to 2017. | 2001-2017 | Notification / Country | Only Pulmonary |
| **S50**  **(68)** | ALMEIDA et al., 2022  Cuba | To spatially categorize children under 15 with tuberculosis in western Cuba between 2011 and 2015 in accordance with the evolving objectives for the eradication of the disease. | 2011-2015 | Notification / Region | Pulmonary and extra pulmonary |
| **S51**  **(69)** | LI et al., 2021  China | To identify notifiable respiratory infectious diseases' spatial distribution and epidemic traits. | 2005-2014 | Notification / Region | Only pulmonary |
| **S52**  **(16)** | ALENE et al., 2021  China | To analyze the spatial distribution of TB in Hunan Province, China, and to determine its socioeconomic, demographic, and environmental determinants. | 2013 to 2018 | Notification / Province | Only pulmonary |
| **S53**  **(70)** | DISMER et al., 2021  Haiti | To use routine TB surveillance data to conduct geographical analysis to see if there were operational geographic levels of TB transmission foci in the Ouest department from 2011 to 2016 that would help PNLT target preventative and control activities. | 2011-2016 | Notification / Country | Pulmonary and extra pulmonary |
| **S54**  **(71)** | KIANI et al., 2021  Iran | To determine the TB incidence rate's spatiotemporal pattern in Iran between 2008 and 2018. | 2008-2018 | Notification / Country | Pulmonary and extra pulmonary |
| **S55**  **(72)** | GIACOMET et al., 2021  Brazil | To determine whether seasonal variation in tuberculosis occurs, to determine the temporal trend of tuberculosis incidence following the deployment of the rapid molecular test (RMT-TB), to categorize the region according to case density and risk regions in Macapá, Amapá. | 2001-2017 | Notification / City | Pulmonary and extra pulmonary |
| **S56**  **(73)** | TITOSSE et al., 2020  Mozambique | Perform a space-time characterization of Maputo, Mozambique's TB prevalence. | 2011-2016 | Notification / Region | Pulmonary and extra pulmonary |
| **S57**  **(74)** | SILVA et al., 2021  Brazil | For the purpose of disease prevention, to identify spatial and spatiotemporal agglomerations of tuberculosis in a priority municipality in northern Brazil. | 2009 to 2018 | Notification / City | Only Pulmonary |
| **S58**  **(75)** | LEAL et al., 2019  Brazil | Examine the spatial distribution of new tuberculosis cases in relation to the locations of the Primary Health Care (PHC) facilities that handled the mandatory notification. | 2010-2014 | Notification / City | Only Pulmonary |
| **S59**  **(76)** | SANTOS et al., 2019  Brazil | Identify potential regions of underreporting or high transmission risk by analyzing the spatial distribution and trend of tuberculosis in the state of Alagoas from 2010 to 2015. | 2010-2015 | Notification / Region | Pulmonary and extra pulmonary |
| **S60**  **(77)** | SILVA et al., 2018  Brazil | To pinpoint determinants impacting the geographic distribution and prevalence of tuberculosis in Olinda, Pernambuco, between 1991 and 2010. | 1991 to 2010 | Notification / City | Only Pulmonary |
| **S61**  **(78)** | CUI et al., 2019  China | To identify the spatiotemporal pattern of tuberculosis notification rates from 2010 to 2016 in the Guangxi Zhuang Autonomous Region of China and any potential relationships with ecological environmental factors. | 2010 to 2016 | Notification / Region | Pulmonary and extra pulmonary |
| **S62**  **(79)** | WANG et al., 2019a  China | From 2010 to 2014, the spatiotemporal patterns and geographical variations of the prevalence of tuberculosis in Inner Mongolia were examined, and associated climatic factors were found. | 2010 to 2014 | Notification / Country | Only Pulmonary |
| **S63**  **(80)** | MAO et al., 2019  China | To analyze epidemic characteristics, spatial auto-correlation, and advanced space-time scan statistics to examine the dynamics of the temporal trends and spatial patterns of smear positive PTB cases at the province level from 2004 to 2015. | 2004-2015 | Notification / Country | Only Pulmonary |
| **S64**  **(81)** | KHAZAEI et al., 2019  Iran | Investigated the spatial and geographic distribution of TB in the province of Hamadan between 1992 and 2013. | 1992-2013 | Notification / Region | Pulmonary and extra pulmonary |
| **S65**  **(82)** | SAMADZADEH et al., 2019  Iran | To find out how TB is disseminated and where hot and cool TB areas have been in Iran's Ardabildo region over the past 12 years. | 2007-2017 | Notification / Region | Pulmonary and extra pulmonary |
| **S66**  **(83)** | TADESSE et al., 2018  Ethiopia | To Identify the location, size and risk of purely spatial and spatiotemporal clusters for high occurrence of tuberculosis in the Gurage Zone, southern Ethiopia during 2007 to 2016 | 2007 to 2016 | Notification / Region | Pulmonary and extra pulmonary |
| **S67**  **(84)** | GUO et al., 2017  China | To inquire how TB differs in different regions and study years in terms of spatial clustering and seasonal variability. | 2005-2013 | Notification / Country | Only Pulmonary |
| **S68**  **(18)** | FAHDHIENIE et al., 2022  Indonesia | To examine if TB clusters exist in this district and their temporal pattern of distribution over the past three years (2019–2021). | 2019-2021 | Notification / Region | Pulmonary |
| **S69**  **(85)** | BIE et al., 2021  China | Use the INLA algorithm and the spatiotemporal distribution model to analyze the spatiotemporal patterns of the relative risk (RR) of tb in mainland China and the impact of seven influencing factors. | 2013-2015 | Notification / Country | Pulmonary and extra pulmonary |
| **S70**  **(86)** | MESQUITA et al., 2022  Brazil | to examine the regional and temporal dynamics of TB in the context of social inequality in northern Brazil from 2001 to 2016. | 2011 and 2015 | Notification / Region | Pulmonary and extra pulmonary |
| **S71**  **(87)** | CASTRO et al., 2018  Brazil | Analyze the effectiveness of TB epidemiological surveillance and describe the social drivers of TB incidence in Manaus as well as its spatial distribution. | 2008-2013 | Notification / City | Only Pulmonary |
| **S72**  **(88)** | WANG et al., 2019b  China | NOT MENTIONED. | 2013-2016 | Notification / Country | Only Pulmonary |
| **S73**  **(89)** | [DIEFENBACH-ELSTOB](https://pubmed.ncbi.nlm.nih.gov/?term=Diefenbach-Elstob%20T%5BAuthor%5D) et al., 2019  Papua, New Guinea | To identify TB patient clusters and characteristics linked to high rates of TB by describing the spatial distribution of the disease in the Balimo District Hospital (BDH) catchment area. | 2013-2017 | Notification / Country | Pulmonary and extra pulmonary |
| **S74**  **(15)** | LIMA et al., 2019  Brazil | The study's goal was to examine the regional and temporal dynamics of TB in northeast Brazil's socioeconomic inequality between 2001 and 2016. | 2001-2016 | Notification / Region | Only Pulmonary |
| **S75**  **(90)** | HUANG et al., 2017  China | Investigating the spatio-temporal dynamics of PTB cases in Zhaotong in order to produce valuable data that will aid decision-makers in creating efficient regional prevention and control strategies. | 2011-2015 | Notification / Region | Only Pulmonary |
| **S76**  **(91)** | ZHANG et al., 2023  China | For targeted TB epidemic intervention, this study examined the temporal and spatial distribution patterns of PTB in Hubei Province. | 2011-2021 | Notification / City | Only Pulmonary |
| **S77**  **(92)** | RAO et al., 2017  China | The objective of this research is to identify the spatial patterns of tuberculosis in the Qinghai province, which may be useful for the formulation and application of important preventative measures. | 2009-2016 | Notification / Region | Only Pulmonary |
| **S78**  **(93)** | DAO et al., 2022  Vietrnam | To provide an online geospatial platform that will aid healthcare professionals in data visualization, active case surveillance in the community, and spatial and temporal TB incidence prediction. | 1st of January 2020 to the 30th of April 2022. | Notification / Country | Pulmonary and extra pulmonary |
| **S79**  **(94)** | MILAHAM et al., 2022  Nigeria | Examined how TB Case Notification Rates, diagnoses, and coverage vary geographically across LGAs. | 2017-2019 | Notification / Region | Pulmonary and extra pulmonary |

Source: Prepared by the author.

**Table S3 -** Spatial analysis methods and the results of the studies included on the systematic review of the geo-spatial hotspots of tuberculosis in the global general population.

| **Study** | **Type of map** | **Cluster detection method** | **Regression statistics method** | **Smoothing technique** | **Results** |
| --- | --- | --- | --- | --- | --- |
| **S1**  **(19)** | Point map  Thematic map  Risk map | Spatial scan statistic  Outro- inverse distance weighted idw-geostatistical approach | Not mentioned | Not mentioned | TB Hotspot – located between Shah Alam hospital and PKNS flat (0 to 0.5 risk  Characteristics –TB burden not directly proportionate with lifestyle status factors factors only a minimal significant correlation occurred during geospatial analysis. |
| **S2**  **(20)** | Rate map | Moran’s I, GetisOrd statistic | Not mentioned | Not mentioned | TB Hotspot –hot spots were localized in the northwest region of the country  Characteristics – Sputum positive TB changed to extra pulmonary TB with a downward trend ratio over 1982–2019. |
| **S3**  **(21)** | Thematic map  Map of temporal tendency | Not mentioned | Regression models (with or without including spatial terms) | Not mentioned | TB Hotspot –four named municipalities had very high TB incidence  Characteristics – The strategic location of the city facilitated tourism attraction |
| **S4**  **(22)** | Point map  Thematic map | k-NN (nearest neighbourhood test) | regression models (with or without including spatial terms) | Not mentioned | TB Hotspot – Most cases were found in a particular village and, the TB distribution showed a trend of clustering with the nearest neighbor index established in the study  Characteristics – High proportion of underprivileged families within the population |
| **S5**  **(23)** | Rate map/ map of temporal tendency | Spatial scan statistics | Not mentioned | Not mentioned | TB Hotspot – The region southeast of Chongqing, which included three counties, had the most likely cluster.  Characteristics - The PTB notification rates in Chongqing were not dispersed randomly. These counties and districts are the most crucial for TB control in the upcoming years because it was shown that they had an excess burden of PTB and had a higher risk of disease transmission. |
| **S6**  **(24)** | Thematic map | Moran’s I, GetisOrd statistic | Bayesian CAR models | Fully Bayesian | TB Hotspot –Concentrated at the county level.  Characteristics –TB notification rate suggested that neighboring counties were more likely to interact with each other |
| **S7**  **(25)** | Thematic map | Moran’s I, GetisOrd statistic | Not mentioned | Not mentioned | TB Hotspot – The Harare city's west south-west region was the hotspot's highest point.  Characteristics – Non-random TB occurrence |
| **S8**  **(26)** | Point Map | Not used (Time series study) | Prais-Winsten Regression Model | Not mentioned | TB Hotspots- Time series analysis  Characteristics - reduction in TB incidence rates in Santa Catarina  Yearly, there was a TB Statistically significant decline in the prevalence of tuberculosis among women |
| **S9**  **(27)** | Rate map | Kulldorff’s scan statistics | Not mentioned | Not mentioned | TB Hotspot – the most likely cluster of TB was in Zhenxiong county.and northeast angle of Yunnan,  Characteristics – TB not randomly distributed |
| **S10**  **(28)** | Thematic map | Spatial scan statistic | Not mentioned | Not mentioned | TB Hotspot – High–High cluster in Jiaoqiao and Changleng towns in the 8-years duration  Characteristics –TB distribution was random across all towns from 2010 to 2018. Higher Tb rates in rural areas than in urban areas. |
| **S11**  **(29)** | Thematic map | None | Gwr | spatial empirical Bayesian | TB Hotspot – The provinces of Xinjiang held the majority of the higher incidence areas.  Characteristics – Obvious geographic heterogeneity |
| **S12**  **(30)** | Kernel density map | Moran’s I, GetisOrd statistic | Not mentioned | Not mentioned | TB Hotspot – located in 4 neighborhoods; NiteróiA, Estância Velha/Olaria and Mathias VelhoB.  Characteristics –TB cases showed heterogeneity across the 29 neighborhoods, |
| **S13**  **(31)** | Rate map | Moran’s I, GetisOrd statistic | Geographically Weighted Regression | Not mentioned | TB Hotspot - Six high-high clusters were detected  Characteristics- Between 2005 and 2015, China's TB incidence was geographically imbalanced, with a high rate in the west and a low rate in the east. |
| **S14**  **(32)** | Thematic map | Morans i, getisord statistic | Regression models (with or without | Not mentioned | high prevalence of respiratory tuberculosis and prevalence in three cities |
| **S15**  **(33)** | Thematic map | Kulldorff’s space-time scan statistical analysis | spatial scan statistic | Not mentioned | TB Hotspots- was located southwest of Xinjiang Uygur  Characteristics- clustering similar in each year |
| **S16**  **(34)** | Rate map | Global moran (lisa) statistics. | Regression models (with or without including spatial terms)  Multilevel poisson regression | Empirical Bayesian | TB Hotspots - On the state's south coast, there was a notable concentration of areas with high TB incidence rates.  Characteristics- Greater TB burden in large metropolitan areas |
| **S17**  **(35)** | Thematic map |  | Not mentioned | Not mentioned | TB Hotspots- risk of extra pulmonary occurrence higher in the western part  Characteristics- Areas with high prevalence had effect on neighboring areas |
| **S18**  **(36)** | Point Map | Kernel point density, | Not mentioned | Not mentioned | TB Hotspots-- The South and Southeast parts of the municipality had the largest concentration of cases.  Characteristics - non-random distribution of TB Cluster detected in high urban areas with social vulnerability |
| **S19**  **(37)** | Thematic map | Morans I, Index | multivariate  regression model, spatial lag models | Fully Bayesian | TB Hotspots- The three clusters detected.  Characteristics- -those who have trouble getting healthcare and are thus more disadvantaged make up the majority of the disease's victims. |
| **S20**  **(38)** | Map of temporal tendency | Moran’s I, GetisOrd statistic | Bayesian car models | spatial empirical Bayesian | TB HOTSPOTS – TB hotspots aggregated in seven quarters within Kerbala Province  CHARACTERISTICS Likely presence of unknown environmental and socio-economic |
| **S21**  **(39)** | Rate map, thematic map, Spatial contiguity matrix map. | Moran’s I, GetisOrd statistic | Not mentioned | spatial empirical Bayesian | TB Hotspots- in the western provinces and in the northern region  Characteristics- Hotspots common along the borders with neighboring countries |
| **S22**  **(40)** | Point map/ Rate map/ Kernel density map | Moran’s I, GetisOrd statistic | Spatial lag models | spatial empirical Bayesian | TB Hotspots- detected in Riehl, Kalk and Mülheim  Characteristics- located closer to the city Centre. |
| **S23**  **(41)** | Density Kernel map | spatial scan statistic Isotonic Scanning | Not mentioned | Spatial empirical Bayesian | TB Hotspots- located in the central region of the municipality  Characteristics- Areas with high demographic density and poor sanitary and socioeconomic conditions had higher TB cases |
| **S24**  **(42)** | Map of temporal tendency | spatial scan statistic | Spatial lag models | Spatial empirical Bayesian | TB Hotspots- located in the northwest regions and southeast rural regions  Characteristics- Decreasing temporal trend. |
| **S25**  **(43)** | Map of temporal tendency | spatial scan statistic | Spatial lag models | spatial empirical Bayesian | TB Hotspots- the Labuhan Ratu, Kedaton, Way Halim, and Sukarame subdistricts were where the most likely cluster was discovered.  Characteristics- All of the clusters shared the same social determinant traits. |
| **S26**  **(44)** | Point map | Not applied | Spatial lag models | NOT MENTIONED | According to the study, socioeconomic characteristics including working, being a housewife, having a low education, smoking, and eating more conventionally are linked to the chance of developing tuberculosis. |
| **S27**  **(45)** | Rate map, Thematic map | Moran’s I, GetisOrd statistic | regression models (with or without including spatial terms) | Spatial empirical Bayesian | TB Hotspots- China southern region from 2004 to 2008,  Characteristics- These areas might have imported TB from a neighboring nation with a high prevalence, such the Philippines. |
| **S28**  **(46)** | Rate Map  Thematic map | spatial scan statistic | regression models (with or without including spatial terms) | spatial empirical Bayesian | TB Hotspots- eight clusters  Characteristics- Living in a rural area, being far from medical facilities, and having inadequate access to medical facilities |
| **S29**  **(47)** | Rate Map  Thematic map | Moran’s I, GetisOrd statistic | regression models (with or without including spatial terms | Not mentioned | TB is not distributed evenly, with a lower incidence in the south-central regions. Eastern regions of the country have the highest rate. |
| **S30**  **(48)** | Rate map | Moran’s I, GetisOrd statistic | Not mentioned | Spatial empirical Bayesian | TB Hotspots- trend toward more TB cases in the south, with seven important clusters. Deep within the rugged regions,  Characteristics- Low socioeconomic position and restricted geographic access to healthcare are two characteristics of this group. |
| **S31**  **(49)** | Map of temporal tendency | spatial scan statistic | Not mentioned | Not mentioned | TB Hotspots- the southern region of Qingdao City and Liaocheng City, where they are concentrated  Characteristics- regional variations in temporal trends of TB prevalence. |
| **S32**  **(50)** | Rate map  Thematic map | spatial scan statistic | Not mentioned | Not mentioned | TB Hotspots - In the Southern and Northern areas of the city.  Characteristics- fewer adults with more schooling and lower capital toward housing |
| **S33**  **(51)** | Thematic map | Moran’s I, GetisOrd statistic | regression models (with or without including spatial terms) | Not used | TB Hotspots- –north-west part of Morocco  Characteristics- poverty and poor housing conditions |
| **S34**  **(52)** | Rate map/ Thematic map | Moran’s I, GetisOrd statistic | GWR | spatial empirical Bayesian | TB Hotspots- central-east part of Lima.  Characteristics- Socio-economic and environmental risk factors of TB incidence |
| **S35**  **(53)** | Thematic map | Moran’s I, GetisOrd statistic | Not specified | Spatial empirical Bayesian | TB Hotspots- in the eastern-central region  Characteristics- TB prevalence spatial heterogeneity, underlying population care-seeking patterns, and access restrictions to diagnostic and treatment facilities. |
| **S36**  **(54)** | Thematic Map | Moran’s I, GetisOrd statistic | mixture models | Not mentioned | TB Hotspots- Rural Ethiopia  Characteristics- Underdevelopment |
| **S37**  **(55)** | Rate map  Thematic map | spatial scan statistic | spatial lag models | Not mentioned | TB Hotspots- central, southern and western part of the country.  Characteristics- congestion caused by formal and informal mining operations, excessive population. |
| **S38**  **(56)** | Map of temporal tendency | Moran’s I, GetisOrd statistic | Not mentioned | Not mentioned | TB Hotspots- Addis Abeba, Adama, Dire Dawa, Bahir Dar, and Shashemene are among the most urbanized regions.  Characteristics- urbanization and population density |
| **S39**  **(57)** | Rate map  Thematic map | Moran’s I, GetisOrd statistic | regression models (with or without including spatial terms) | spatial empirical Bayesian | TB Hotspots- During the time of the research, in the continental US.  Characteristics- economic factors, proportion of population of all ages in poverty  economic factors, the percentage of people of all ages who are poor |
| **S40**  **(58)** | Rate map  Thematic map | Moran’s I, GetisOrd statistic | regression models (with or without including spatial terms) | Spatial empirical Bayesian | TB Hotspots- The eastern region of Korea is where hotspots are mostly found.  Characteristics- Migration |
| **S41**  **(59)** | Thematic map | Moran’s I, GetisOrd statistic | Regression models (with or without including spatial terms) | Spatial empirical Bayesian | TB Hotspots- Mirab Armacho, Tach Armacho, Metema and Tsegede  Characteristics – A weak TB control program in the area, insufficient availability of TB treatment and diagnosis, and poor treatment adherence. |
| **S42**  **(60)** | Rate map  Thematic map  Map of central tendency | Moran’s I, GetisOrd statistic | regression models (with or without including spatial terms) | Spatial empirical Bayesian | TB Hotspots ‐ prison in the southwest section of Gombak near Rawang mukim  Characteristics - socioeconomic and environmental influences on TB cases. |
| **S43**  **(61)** | Rate map  Thematic map | Spatial scan statistic | Mixture models | Spatial empirical Bayesian | TB Hotspots- high-risk zones accounting 22% |
| **S44**  **(62)** | Map of temporal tendency | Moran’s I, GetisOrd statistic | GWR  regression models (with or without including spatial terms) | Not mentioned | TB hotspots- located in the northwest and south  Characteristics - Temperature, humidity and precipitation were related to TB incidence. |
| **S45**  **(63)** | Point map  Rate map  Thematic map  Map of temporal tendency | Not specified | regression models (with or without including spatial terms) | Not mentioned | TB Hotspots- In four districts of Punjab which are Bahawalpur, Faisalabad, Gujranwala and Lahore  Characteristics –Unbalanced population socioeconomic factors |
| **S46**  **(64)** | Point map | Moran’s I, GetisOrd statistic | regression models (with or without including spatial terms) | Not mentioned | TB Hotspots- TB incidence and the percentage of clustered TB cases have a modest correlation.  Characteristics - residents of low-transmission areas might travel often to regions where there is a high risk of contracting tuberculosis (TB), such as those where there is a high prevalence of HIV, malnutrition, or the use of biomass fuel. |
| **S47**  **(65)** | Rate map | Moran’s I, GetisOrd statistic | Mixture models | Not mentioned | TB Hotspots- municipalities classified as High–High (high rates of COVID with high rates of TB).  Characteristics - increasing temporal trend of the TB notification rate in the pre-pçandemic period, especially in the North |
| **S48**  **(66)** | Thematic map  Rate map | spatial scan statistic | GWR  regression models (with or without including spatial terms | empirical Bayesian | TB Hotspots- -Located in Sobral, a municipality in the state's rural interior, and the metropolitan area of Fortaleza.  Characteristics - The most probable cluster, which exhibits the characteristics of an urban agglomeration—constant migration between towns and greater levels of poverty—is constituted of the state's capital with the towns in the metropolitan region. |
| **S49**  **(67)** | Rate map  Thematic map  Map of central tendency | Moran’s I, GetisOrd statistic  spatial scan statistic | spatial lag models | empirical Bayesian | TB Hotspots- Six of the 15 spatial-temporal clusters are in the state of So Paulo and spread over the northern and Southeastern regions.  Characteristics - social vulnerability, low urban infrastructure and human capital, |
| **S50**  **(68)** | Rate map  Thematic map | Not specified | regression models (with or without including spatial terms) | Not mentioned | TB Hotspots- Not Indicated  Characteristics - Not Indicated |
| **S51**  **(69)** | Rate map  Thematic map  Map of temporal tendency | Moran’s I, GetisOrd statistic  spatial scan statistic | Mixture models | spatial empirical Bayesian | TB Hotspot- Mainly in Binzhou, Liaocheng and Linyi.  .  Characteristics - Not specified |
| **S52**  **(16)** | Rate map  Choropleth map | Moran’s I, GetisOrd statistic | spatial lag models  Bayesian spatial Poisson regression models | fully Bayesian | TB Hotspots- observed in the western part of the province  Characteristics – Climatic factors and health care access |
| **S53**  **(70)** | Point map  Rate map  Thematic map  Map of temporal tendency | Moran’s I, GetisOrd statistic  Spatial scan statistic | Not mentioned | Not used | TB Hotspot- located near the coast of Port-au-Prince, Carrefour, Cité-Soleil, and in the northeastern quartiers of Delmas’. |
| **S54**  **(71)** | Rate map  Thematic map  Map of temporal tendency | Moran’s I, GetisOrd statistic  Spatial scan statistic | Not mentioned | Not mentioned | TB Hotspots- Iran’s northeast and southeast have been designated as TB hotspots.  Characteristics - The province has a high incidence of TB, while nearby areas have a low prevalence. |
| **S55**  **(72)** | Thematic map  Map of temporal tendency  Density kernel map | Moran’s I, GetisOrd statistic  Spatial scan statistic | regression models (with or without including spatial terms) | Not mentioned | TB Hotspots - Central, Northern and Southern districts  Characteristics - low Human Development Index, stilt houses, absence of sanitation, clusters of population, and large number of informal workers corroborate the spread of the disease. |
| **S56**  **(73)** | Map of temporal | Moran’s I, GetisOrd statistic  Spatial scan statistic | Not mentioned | Not mentioned | TB Hotspot - El Salado neighborhood in the North  Characteristics - low socioeconomic sphere and high poverty conditions |
| **S57**  **(74)** | Point map  Thematic map  Map of temporal tendency | Moran’s I, GetisOrd statistic  Spatial scan statistic | Not mentioned | Not used | TB Hotspots- 25 census sectors; the Centro, Mercadinho, Bacuri, Parque Anhanguera, Nova Imperatriz, Vila Lobão, Santa Rita neighborhoods  Characteristics - lower demand for health services in the periphery, as well as the accentuated population agglomeration in the Central region. |
| **S58**  **(75)** | Rate map  Thematic map  Density kernel map | Moran’s I, GetisOrd statistic | regression models (with or without including spatial terms) | Not mentioned | TB Hotspots- south and southwest of Belém  Characteristics - lower socioeconomic conditions |
| **S59**  **(76)** | Rate map | Moran’s I, GetisOrd statistic | regression models (with or without including spatial terms) | empirical Bayesian | TB Hotspots- Eastern Alagoan mesoregion  Characteristics – Precarious access to health services, high degree of vulnerability and under diagnosis. |
| **S60**  **(77)** | Rate map  Thematic map  Map of temporal tendency Thesis/Dissertation | Not mentioned | Not mentioned | Not mentioned | TB Hotspots- In the metropolitan region of São Paulo, municipalities on the south coast of São Paulo  Characteristics - Worse socioeconomic and demographic conditions |
| **S61**  **(78)** | Map of temporal tendency | Moran’s I, GetisOrd statistic  Spatial scan statistic | regression models (with or without including spatial terms) | empirical Bayesian | TB Hotspots- Centrally located in Guangxi  Characteristics - Gross domestic product per inhabitant is a negative measurement of growth in socioeconomic status. |
| **S62**  **(79)** | Rate map  Map of temporal tendency | Moran’s I, GetisOrd statistic | Not mentioned | Fully Bayesian | TB Hotspots- high-high areas spread from Inner Mongolia are northeastern to the southeasterly regions.  Characteristics - Due to poor economic conditions and a lack of public health resources, farming was the profession most at risk for TB., |
| **S63**  **(80)** | Thematic map  Map of temporal tendency | Moran’s I, GetisOrd statistic  Spatial scan statistic | Not mentioned | Not mentioned | TB Hotspots- HH cases clusters moved toward southwest more obviously. |
| **S64**  **(81)** | Thematic map  Map of temporal tendency | Spatial scan statistic | regression models (with or without including spatial terms | Not mentioned | TB Hotspots- Kabudarahang and Famenin counties.  Characteristics- factors such as socioeconomic position, the environment, or medical issues such the prevalence of HIV infection, undernourishment, and diabetes |
| **S65**  **(82)** | Point map  Rate map  Thematic map | Moran’s I, GetisOrd statistic | Not mentioned | Not mentioned | TB Hotspots- the center of the province and in the north of the province  Characteristics - Regular border crossings; the comparatively underprivileged and dense immigrant population |
| **S66**  **(83)** | Point map  Thematic map | Moran’s I, GetisOrd statistic  Spatial scan statistic | Not mentioned | Not mentioned | TB Hotspots- At the edges of the geographical zone, southwest of the Abeshege District  Characteristics of Hotspots- The risk of contracting TB was 4.16 times higher for persons inside this cluster than for those outside. Additionally, 11 significant secondary clusters with a high TB prevalence were found. |
| **S67**  **(84)** | Rate map  Map of temporal tendency  Density kernel map | Moran’s I, GetisOrd statistic | regression models (with or without including spatial terms) | fully Bayesian | TB Hotspots- Northwest; Xinjiang, Qinghai, Tibet, and Yunnan and central China; Hunan.  Characteristics - underdeveloped economic conditions, poor health care, and ignorance |
| **S68**  **(18)** | Rate map  Map of temporal tendency  Density kernel map | Moran’s I, GetisOrd statistic  Spatial scan statistic | Not mentioned | Not mentioned | TB Hotspots- in North Aceh District and the Heureudong Pase sub-district  Characteristics – High population density facilitating transmission of TB and high level of community mobility to the capital city. |
| **S69**  **(85)** | Map of temporal tendency  Density kernel map | Moran’s I, GetisOrd statistic  Spatial scan statistic | regression models (with or without including spatial terms) | fully Bayesian | TB Hotspots- Northwest and South China regions  Characteristics - a year-round tropical marine ecosystem characterized with excessive amounts of humidity, frequent rainfall, and constant daylight. |
| **S70**  **(86)** | Point map  Map of temporal tendency  Density kernel map | Moran’s I, GetisOrd statistic | Not mentioned | empirical Bayesian | TB Hotspots- Sacramenta (Fátima, Telégrafo, Sacramenta, and Barreiro) and Guam (Jurunas, Condor, Guam, and Terra Firme).  Characteristics - the most populated area of Belém, with a large concentration of households with many occupants. highest HDI |
| **S71**  **(87)** | Rate map  Thematic map  Map of temporal tendency | Moran’s I, GetisOrd statistic | regression models (with or without including spatial terms) | Not mentioned | TB Hotspots- south and west of the city  Characteristics - unemployment and poor access to running water. |
| **S72**  **(88)** | Rate map  Map of temporal tendency | Moran’s I, GetisOrd statistic | regression models (with or without including spatial terms) | Not mentioned | TB Hotspots- the western districts, mainly in Xinjiang, Tibet and Qinghai province  Characteristics - located in the interior, with weak economic foundations, poor medical resources and low educational level of residents |
| **S73**  **(89)** | Point map  Thematic map | spatial scan statistic | regression models (with or without including spatial terms) | Not mentioned | TB Hotspots- Lower sections of the Bamu and Gama Rivers are located in the Bamu area.  Characteristics - under diagnosis of TB |
| **S74**  **(15)** | Rate map  Thematic map  Map of temporal tendency | Moran’s I, GetisOrd statistic | regression models (with or without including spatial terms | empirical Bayesian | TB Hotspots- in the Southeast (between 2001 and 2015) and Northeast (between 2001 and 2009) of the state.  Characteristics - poor economic growth whereas there was a larger frequency in more urbanized areas between 2011 and 2015. |
| **S75**  **(90)** | Thematic map  Map of temporal tendency | Moran’s I, GetisOrd statistic | Not mentioned | Not mentioned | TB Hotspotsthe core and Wufeng regions of Zhenxiong  Characteristics - inadequate care, poor treatment, or patient management. |
| **S76**  **(91)** | NO MAP | Moran’s I, GetisOrd statistic  Spatial scan statistic | Not mentioned | Not mentioned | TB Hotspots- Enshi Prefecture, southwest Hubei province  Characteristics - backward economic conditions and inconvenient |
| **S77**  **(92)** | Thematic map  Distance map | Moran’s I, GetisOrd statistic | Not mentioned | Not mentioned | TB Hotspots- southwest of Qinghai  Characteristics - very low income, poorer living conditions and sanitation |
| **S78**  **(93)** | Rate map  Thematic map | Spatial scan statistic (WebGIS) | Not mentioned | Not mentioned | TB Hotspots - southwestern part of the  country. namely Ha Noi, Da Nang, and Ho Chi Minh City  Characteristics - TB patients’ movement to major cities |
| **S79**  **(94)** | Rate map  Choropleth map | Global Moran’s  Index (Global Moran’s I) | Not mentioned | Not mentioned | TB Hotspots- In Local Government Areas (Jibia, Kurfi, and Batagarawa) that are adjacent to or surround the state capital  Characteristics – Due to an inadequate pattern of the Xpert facility distribution, the local government areas on the state's boundary of Sokoto State and the Niger Republic were not covered by the diagnostic services which were provided. |

Source: Prepared by author.

**Table S4 -** Synthesis of TB hotspots characteristics throughout the world found in the studies included on the systematic review of the geo-spatial hotspots of tuberculosis in the global general population. These number corresponds to the study number for the articles selected where S1 is Reference 14 on the list.

|  | - Lifestyle risk ^(S1)^  - Unstable/ non-random cluster ^(S2, 4, 5,7,9,19,33,63,66)^  - Hotspots spread to neighboring cities ^(S3,4,6,12,18,23,29,54,63,70,76)^  - Hotspot related to tourism ^(S3)^  - Climate related clustering ^(S4,13,28,36,41,44,46,54,66,67,71,74,78)^  - Poor socioeconomic conditions ^(S4,12,13,19,20.24,25,28,32,34,35,36,41,44,47,49,50,51,54,57,58,60,63,64,67,69,72,73,74,75,78,79)^  - High population density ^(S17,24,39,40,50,57,58,60,61,70,72,75,76,77)^  - Poor health facility/ service/distance ^(S30)^  - Race related hotspots ^(S41)^ |
| --- | --- |

Source: Prepared by author.
